# Phytochemical Profiles of Plant Materials: From Extracts to Added-Value Ingredients

**DOI:** 10.3390/plants13070964

**Published:** 2024-03-27

**Authors:** Lina Raudone, Nijole Savickiene

**Affiliations:** 1 Department of Pharmacognosy, Lithuanian University of Health Sciences, Sukileliu Av. 13, 50162 Kaunas, Lithuania; nijole.savickiene@lsmu.lt; 2 Laboratory of Biopharmaceutical Research, Institute of Pharmaceutical Technologies, Lithuanian University of Health Sciences, Sukileliu Av. 13, 50162 Kaunas, Lithuania

In the scientific research on medicinal and food plants, studying phytochemical profiles in plant materials has gained increasing attention over the years [1]. Plants primarily generate secondary metabolites, such as phenolics, terpenes, and organic acids, as a defense mechanism against environmental challenges. Interestingly, these compounds also have health-promoting benefits for humans and animals [2,3]. Neglected and underutilized plants have gained significance in the food and pharmaceutical industries as viable alternatives to synthetic nutraceuticals and chemicals; however, their full potential remains unknown. Research on these overlooked plant resources is crucial to uncover innovative sources for natural nutraceuticals, antioxidants, and added-value ingredients [4]. This exploration facilitates a transition from basic extracts to the creation of added-value products, thereby unlocking the full potential of plant materials. Paradigmatic shifts from raw extracts to added-value ingredients are marked by focusing on standardization, quality control, and innovation. Standardizing the phytochemical content ensures consistency and efficacy, while rigorous quality control measures safeguard against contaminants [5]. Concurrently, integrating innovative technologies, such as nanotechnology and encapsulation, facilitates the development of novel delivery systems, improving the bioavailability of phytochemicals and expanding their applications [6,7]. The preparation of reproducible extracts starts within the optimal harvesting time, optimized extraction conditions and targeted plant part selection for investigation. Developing value-added ingredients opens avenues for market expansion, fostering economic growth in the plant-based products sector [5]. However, it is crucial to address sustainability concerns, ensuring ethical sourcing and production practices to mitigate environmental impacts and promote long-term viability.

The study by Radusiene et al. investigated the root phenolic profiles of five *Solidago* species, including native and alien varieties, aiming to uncover the potential applications and botanical origins of these species (Contribution 1). The roots of *S. virgaurea*, *S. gigantea*, and *S. ×niederederi* exhibited a statistically similar phenolic content, while *S. canadensis* and *S. ×snarskisii* displayed lower compound levels. The analysis of radical-scavenging capacities revealed the potential health benefits of these root extracts. This study provides valuable insights into the phenolic composition of *Solidago* species, offering potential applications across diverse fields. Converting invasive species into added-value ingredients holds significant potential for sustainable resource utilization [8]. By exploring the phytochemical profiles of these invasive species, valuable bioactive compounds can be identified and extracted, contributing to the development of novel products in industries such as pharmaceuticals, cosmetics, and functional foods [9]. This approach mitigates invasive species’ ecological impact and transforms them into valuable resources, fostering economic growth and innovation.

Ivanauskas et al. investigated the dynamic changes in the composition of phenolic compounds and triterpenoid saponins in *Epilobium angustifolium* leaves and flowers across different flowering phases (Contribution 2). Researchers identified 13 polyphenolic compounds and seven triterpenoids in the plant material. This study highlights how, while the optimal content and profile of polyphenols occur during late flowering, triterpenoids reach their maximum levels during mass flowering, emphasizing the potential of *E. angustifolium* raw material as a source of oenothein B and triterpenoids. Selecting the proper harvesting time is paramount for maximizing the quality and quantity of bioactive compounds in plant materials, thereby ensuring the production of added-value ingredients with enhanced therapeutic efficacy and commercial potential.

The study by Di Giacomo et al. highlights the importance of exploring the potential of hemp bioproducts from specific varieties cultivated in the Lazio Region for nutraceutical and pharmaceutical applications (Contribution 3). This research elucidates the diversity of bioactive compounds in hemp inflorescences by conducting a comprehensive analysis of chemically characterized hydroalcoholic extracts. The investigation’s focus on antimutagenic activities, cytotoxicity, oxidative stress modulation, and radical scavenging properties provide valuable insights into the chemo-preventive potential of these hemp varieties, suggesting their viability as a source of bioactive compounds for preventive or adjuvant healing strategies.

Galinytė et al. raise concerns regarding the relevance of assessing heavy metal safety in naturally collected cyanobacterial biomasses for potential pharmaceutical applications of cyano-phycocyanin (Contribution 4). This biliprotein with antioxidative, anti-inflammatory, and anticancer properties in cyanobacteria can be sustainably transferred from waste to added-value ingredients. Still, scientific uncertainties remain regarding the levels of toxic metals and research on suitable waste resources. 

Certain common plant species with well-established medicinal usage still have their underutilized side. Hordiei et al. focus on the phenolic profiles and antioxidant activity of cultivated *Tanacetum parthenium* (feverfew), a plant rich in bioactive compounds (Contribution 5). Identified phenolic compounds, particularly hydroxycinnamic acids such as chlorogenic acid, highlight feverfew’s potential anti-inflammatory and antioxidant properties. This study highlights the utilization of Ukrainian feverfew as a valuable raw material for developing herbal medicines, emphasizing the relevance of quality assessment in determining pharmacological activity. Furthermore, the applicability of *Tanacetum* genus plants is advocated by the research team of Šukele et al. (Contribution 9). They characterized the phenolic compounds, thujone content, and antioxidant activity of *Tanacetum vulgare* L. (Tansy) harvested from different regions of Latvia, providing valuable insights into its chemical profile. The research highlighted significant variations in the extraction yield, total phenol content, and antioxidant activity among collection regions and aerial parts, emphasizing the potential of *T. vulgare* as a source for herbal products. Detecting thujone content targets the importance of assessing toxic constituents, contributing to this study’s relevance from both medicinal and product development perspectives. Indeed, investigating native herbs and fruits is important due to their ecological adaptation, which often results in unique and specialized compounds offering distinctive flavors, nutritional benefits, and therapeutic properties, contributing to sustainable and culturally relevant solutions [10]. Their extracts as valuable ingredients enhance the diversity and richness of formulations in various industries, such as cosmetics, food, and pharmaceuticals. Ali et al. researched native Australian plants *Mentha australis*, *Mentha satureioides*, *Apium prostratum* and *Solanum centrale* (Contribution 10). Their findings, including identifying and semi-quantifying key compounds and predicting pharmacokinetic properties, can elucidate scientific uncertainties that need solving in further research into the nutraceutical and phytopharmaceutical potential of these native plants.

Underutilized plant species may possess essential nutrients, but studies on their composition and consumption are limited and fragmented. Information about phytochemical, nutritional and functional characteristics is important to promote and expand the utilization of these plant materials, enhancing ingredient security [11]. Konaré et al. investigated microencapsulation as a method for delivering bioactive compounds from neglected and underutilized species (*Balanites aegyptiaca* and *Ziziphus mauritiana*) using a combination of whey protein, pectin, soy protein, and maltodextrin (Contribution 13). The encapsulation process resulted in high yields and efficiencies, and in vitro gastrointestinal tests demonstrated the enhanced bioavailability of bioactive compounds. Analysis revealed significant levels of bioactive metabolites showcasing the antidiabetic, anti-inflammatory, and antioxidant effects of the microencapsulated compounds, emphasizing the potential of these fruits for application in the food and pharmaceutical industries. González-Peña et al. reviewed the properties and applications of carotenoids in the frame of protecting their unstable chemical properties by applying encapsulation techniques and enhancing bioavailability (Contribution 15). Developing products and technologically functionalized, underutilized plant materials can establish a direction for future scientific research. 

The extraction process is a key step for preparing high-quality extracts from botanical matrices [12]. It is important to consider variables such as extraction solvent, temperature, and time to optimize extraction parameter selection. Optimization studies enable the identification of optimal conditions that maximize extraction efficiency while maintaining the desired chemical profile of the plant extract [13]. Setyani et al., utilizing a Box–Behnken design under a response surface methodology (RSM), investigated the ultrasound-assisted extraction conditions for *Moringa oleifera* (Contribution 6). By focusing on four critical factors—solvent ratio, solvent–solid ratio, extraction temperature, and extraction time—this study efficiently optimized the preparation of plant extracts. The identified optimal conditions, including a 50% *v*/*v* solvent ratio, 30% *v*/*w* solvent–solid ratio, 35 °C extraction temperature, and 45 min extraction time, were substantiated through both experimental and predicted values, highlighting the precision and reliability of the applied RSM model in achieving optimal extraction parameters. The study by Taraseviciene et al. explored the chemical composition and extraction efficiency of stinging nettle (*Urtica dioica* L.) in Lithuania, emphasizing the impact of factors like the growing region, soil, and meteorological conditions on the plant’s antioxidant activity (Contribution 12). The results reveal the phytochemical peculiarities of leaves and roots. This study also explored maximizing the extraction efficiency of bioactive compounds from stinging nettle, which is crucial for fully exploiting the plant’s potential in various applications. The strategic differentiation of plant parts in food processing is a prerequisite for sustainable and zero-waste strategies. Resource efficiency can be maximized using various plant parts such as the stems, leaves, roots, and seeds. This targeted approach minimizes waste and unlocks each plant part’s full nutritional and functional potential, contributing to a more sustainable and economically viable food production chain [5]. The findings of Ispiryan et al. indicate significant variations in the sugar content, mineral composition, phenolic content, and antioxidant activity among different plant parts, highlighting the potential of raspberry plant parts as valuable sources of natural food ingredients with health-promoting properties, offering economic attractiveness for consumers and emphasizing the relevance of precise differentiation in production processes (Contribution 7).

The selection of plant varieties with improved resistance to climate change and adaptation to ecological conditions is crucial for ensuring food security and agricultural sustainability in response to changing environmental patterns. These resilient plant varieties enhance crop yields and farmers’ livelihoods and contribute to the overall resilience of ecosystems, fostering biodiversity and mitigating the impact of climate-induced challenges on global food production [14]. Kalasuba et al. reviewed the plant *Rhizophora stylosa*, revealing its phytochemical, pharmacological diversity, and ecological significance (Contribution 14). Fougère et al. provided a detailed phytochemical analysis of corn silk extracts, identifying 104 molecules (Contribution 11). Their research mapped the corn silk extract’s composition, highlighting the homologous series of lipids, monoglycosylated flavonoids, diglycosylated flavonoids, and organic acids. These findings have relevance in enhancing the selection of corn varieties with improved resistance or bioactive properties, contributing to a deeper understanding of the plant’s defense mechanisms and growth factors.

Phenolic compounds play a crucial role in the production of silver nanoparticles by acting as effective reducing and stabilizing agents. These compounds, which are abundant in plant extracts, facilitate a reduction in silver ions, promoting the formation of stable particles [15]. Qanash et al. emphasized the importance of medicinal plants in the formation of nanoparticles as a safe and convenient process (Contribution 8). They revealed the potential of plant-derived production in the development of nanotechnologies. The resulting nanoparticles exhibit notable antimicrobial properties, disrupting microbial cell membranes and processes. Additionally, the presence of phenolic compounds contributes to the antioxidant activities of silver nanoparticles, scavenging free radicals and reducing oxidative stress [15]. Plants serve as a sustainable source for silver nanoparticle production due to their accessibility, renewability, and the eco-friendly nature of the synthesis process, making them an environmentally conscious alternative to conventional methods. 
